# Effect of tenofovir containing ART on renal function in patients with moderate/severe reduced creatinine clearance at baseline: A retrospective study at two referral hospitals in Namibia

**DOI:** 10.1002/prp2.681

**Published:** 2022-12-30

**Authors:** Francis Kalemeera, Brian Godman, Andy Stergachis, Tim Rennie

**Affiliations:** ^1^ Faculty of Health Sciences University of Namibia Windhoek Namibia; ^2^ Strathclyde University Karolinska Institute Sefako Makgatho University of Health Sciences Ga‐Rankuwa South Africa; ^3^ School of Pharmacy University of Washington Seattle Washington USA

**Keywords:** Creatinine Clearance, Decline, HIV, Improvement, Tenofovir Disoproxil Fumarate

## Abstract

Prescription of tenofovir disoproxil fumarate (TDF) for patients with baseline creatinine clearances (CrCl) <60 mL/min is said to increase risk of further decline in CrCl. Study objectives were to assess incidence of improvement and predictors thereof; to assess incidence of decline and transition to lower stages of CrCl; and comparison of declines between patients with a baseline CrCl < 60mL/min (group‐I) and ≥ 60 mL/min (group‐II). The study was retrospective, included patients 16 yrs or older who received TDF‐containing ART. Improvement and decline were defined as ≥ 25% increase or decrease in CrCl, respectively. Binary logistic regression was performed to identify predictors of improvement. Groups I and II had 2862 and 7526 patients, respectively. In group‐I, improvement in CrCl was observed in 40.1% (n = 1146), and was associated with stage IV of CrCl (adjusted Odds Ratio [aOR]=13.4 [95% CI: 6.7 ‐ 26.9, *P* < .001]); male gender (aHR = 1.8 [95% CI: 1.5 ‐ 2.2, *P* < .001]); and a poor HIV‐status (aHR = 1.2 [95% CI: 1.0 ‐ 1.4], *P* = .033). In group‐I and group‐II, respectively, decline occurred in 2.3% and 13.0%, (*P* < .001); transition to lower stages occurred in 1.0% and 25.2% (*P* < .001); and migration to stage IV CrCl occurred in 1.0% and 0.5% (*P* < .001). Improvement was more likely than decline in group‐I patients. Although, group‐I patients were more likely to experience new onset severe reduced CrCl than group‐II patients, the proportions were extremely low. TDF should not be withheld from HIV‐positive patients with a baseline CrCl < 60 mL/min.

## Graphical abstract

Panel A shows median CrCl values for patients in the four stages of CrCl at baseline. Panel B shows the follow‐up median CrCl for the same baseline stages. A drop is observed for stages 1 and 2, while increases are observed for stages 3 and 4.

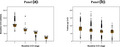

## 1 BACKGROUND

During the pre‐antiretroviral therapy (ART) era, HIV‐related renal impairment was a relatively frequent morbidity and cause of mortality, especially in patients of African descent.^1^, ^2^, ^3^ However, the frequency of HIV‐related renal impairment, was appreciably reduced when ART was introduced.^4^, ^5^ Nowadays, ART naïve patients who present with moderate or severe reductions in renal function are likely reporting late for treatment and care, or are poorly adherent to ART.^6^ ART has been shown to improve renal function in these patients.^7^, ^8^, ^9^ From 2010, in Namibia, to the time of writing this manuscript, tenofovir disoproxil fumarate (TDF)–containing ART regimens have been preferred over other regimens because of TDF’s better safety profile.^10^, ^11^, ^12^, ^13^ Nevertheless, TDF is an independent risk factor for nephrotoxicity.^9^, ^14^, ^15^ Because of TDF‐related nephrotoxicity, Namibia's 2016 ART guidelines recommended the prescription of an alternative to TDF when the HIV‐positive patient had a baseline CrCl < 60 mL/min.^11^ On the contrary, the guidelines allowed the prescription of TDF‐containing ART for patients with a baseline CrCl < 60 ml/min, if they were co‐infected with hepatitis‐B,^11^ which is relatively frequent in Namibia.^16^ The results of a recent study we conducted, albeit in a few patients from one facility, suggested that improvement is expected in HIV‐infected patients who receive TDF containing ART with a baseline CrCl < 60 mL/min.^17^ The primary objective of this study was to confirm, with a larger number of patients, whether the initiation of TDF containing ART was associated with improvement or an increased risk of worsening renal function in patients who had their CrCl in the moderate/severe stage, at baseline. The secondary objectives were to identify the factors that were associated with the improvement in renal function, and to compare the CrCl decline rates between the patients who had a moderate/severe reduced CrCl and those who had mildly reduced/normal CrCl at baseline.

## 2 MATERIALS AND METHODS

### 2.1 Study design and inclusion criteria.

First, this was a retrospective cohort study that included patients who initiated TDF‐containing ART from August 2010 to December 2016, because TDF‐containing ART became the preferred initial ART regimen in Namibia in July 2010.^18^ Second, patients who were 16 years and older at the time of ART initiation were included, as recommended in the guidelines.^18^ Third, patients whose renal function stage was moderate/severe reduced were included for the primary analysis. The secondary analysis was a comparison of the rates of adverse renal outcomes between the patients who had a moderate renal function at baseline, and those who had mild/normal CrCl at baseline. The Namibia ART guidelines recommend the use of Cockcroft‐Gault's equation to calculate CrCl.^18^ The equation is as follows:

$$\begin{array}{c} \text{CrCl}\;=\;\left(140‐\text{age}\left[\text{year}\right]\right)\times \frac{\text{weight}(\text{kg})}{\text{serum creatinine}\;(\mu \text{mole per}L)}\times 1.22 \end{array}$$

For female patients, the value generated from the above equation is multiplied by 0.85. NB: The pharmacists at the health facilities calculate and record the CrCl results in the patient's file (not published). Serum creatinine is measured by the Namibia Institute of Pathology, which has a functional lab at each public health hospital in Namibia.

### 2.2 Setting

The study depended on data from two intermediate referral hospitals: Oshakati Intermediate Hospital, a 750‐bed capacity hospital in Northern Namibia, and Katutura Intermediate Hospital, an 830‐bed capacity hospital in Windhoek, the capital city of Namibia. Together, these hospitals provided HIV treatment and care to over 40,000 patients, according to the data that were accessed from the Ministry of Health and Social Services (MoHSS) for this study.

### 2.3 Study variables

#### 2.3.1 Independent variables

The independent variables included gender, age, weight, follow‐up duration, ART regimen, pregnancy, stage of renal function, and status of HIV infection. Status of HIV infection was dichotomous in nature: undesired status of HIV infection and desired status of HIV infection. Undesired status of HIV infection constituted a CD4 count < 200 cells/mm^3^ or a viral load > 1000 copies/mL or tuberculosis (TB) or a combination of these variables. We used a CD4 count < 200 cells/mm^3^, because this is when opportunistic infections and other HIV‐related diseases escalate and can be an indicator of treatment failure.^19^, ^20^, ^21^ We used a viral load > 1000 copies/ml, because treatment failure is defined as a viral load > 1000 copies/mL at 24 weeks of ART.^22^, ^23^, ^24^ Lastly, we included tuberculosis as it is a known indicator of treatment failure.^25^

### 2.4 Dependent variables (Endpoints).

The endpoints of the study were:

1. Improvement in CrCl
  ▪ Improvement in CrCl was defined as an increase in CrCl by ≥ 25% based on the difference between the baseline and last measured CrCl. Additionally, an increase in CrCl by ≥ 3ml/min/year was investigated.
2. Decline in CrCl
  ▪ Decline in CrCl, was defined as a ≥ 25% reduction in CrCl, relative to the baseline.
3. Transition from baseline to lower stages of CrCl.
  ▪ These included transitions from stages I and II to stages III and IV; and from stage III to stage IV.
  ▪ The stages of CrCl and their limits, were stage I (≥90 ml/min); stage II (60 ‐ 89 ml/min); stage III (30 ‐ 59 ml/min); and stage IV (<30 ml/min)].
4. Transition to stage IV of CrCl.
  ▪ This included from a baseline CrCl ≥ 60 ml/min (stages I and II) to stage IV; and from stage III to stage IV

### 2.5 Data analysis

The characteristics of CrCl < 60 mL/min group were described using means and standard deviations (SD) for continuous variables and proportions for categorical variables. The difference in CrCl between the baseline and the last recorded value was used to determine whether improvement or decline in CrCl had occurred. Then the < 60 mL/min group of patients were sub‐divided into the improvement and non‐improvement groups. The means (SD) and proportions in the improvement and non‐improvement groups were used for descriptive purposes. The proportion of patients who experienced decline in CrCl among the < 60 mL/min group and the corresponding proportion among the ≥ 60 mL/min group were calculated.

For the < 60 mL/min group of patients, we compared the means (SD) and proportions of patients in the improvement group with the corresponding means and proportions in the non‐improvement group. We used the student's t‐test for continuous variables, and Pearson's chi‐square test for categorical variables. To identify the variables that were associated with improvement in CrCl, we performed binary logistic regression analysis. First, we conducted univariate analysis for each independent variable. Then we included all the independent variables in the multivariate analysis model, which we performed using the backward stepwise conditional method. This method eliminates variables from the model based on the likelihood‐ratio statistic on conditional parameter estimates.^26^, ^27^ As such, only variables that met the pre‐set conditions of significance, were left in the final step of the multivariate analysis model.

We conducted sub‐analysis estimates of the mean duration of follow‐up for the improvement and non‐improvement groups for the first four time‐intervals. These time‐intervals were included, because one could confirm that improvement had occurred in the first‐two time intervals (0‐6 months & 7‐12 months), while for the second two time‐intervals (13‐18 months & 19‐24 months), improvement could have occurred in the first two time‐intervals. For each improvement group, we compared the duration of follow‐up for the improvement group and the non‐improvement group, using Levene's test to assess similarity of variances, and the independent students t‐test for the difference means. (NB: The day the last CrCl was recorded served as the end of follow‐up period.) We conducted binary logistic regression sub‐analysis, to compare with the results that were generated for the original analysis.

Next, we compared the proportion of patients who experienced the ≥ 25% decline in CrCl among the < 60 mL/min group with the corresponding proportion among the ≥ 60 mL/min group using the Student's t‐test, and assessed the proportion of patients who experienced a shift to lower stages of CrCl. We evaluated the proportions who shifted to stage IV CrCl between the < 60 and ≥ 60 mL/min groups. (NB: For the group who had a baseline CrCl from 30‐<60 mL/min, the lower stage of renal function was stage IV CrCl: <30 mL/min. For the group who had a baseline CrCl ≥ 60 mL/min the lower stages included: stage III CrCl (CrCl 30‐59 mL/min), and stage IV CrCl (<30 mL/min). All analyses were conducted using SPSS version 22. The *P*‐value was set at < 0.05 and the confidence interval at 95%.

### 2.6 Ethics

The study was approved by the Faculty of Health Sciences, the ethics review board of the University of Namibia, and the MoHSS. The study's reference number at the MoHSS is 17/3/3. There was no contact with the patients, and there was no involvement of health care workers. Anonymity of the patients was assured by eliminating any patient identifier data. The data were only available to the one of the researchers.

## 3 RESULTS

**Figure 1:**
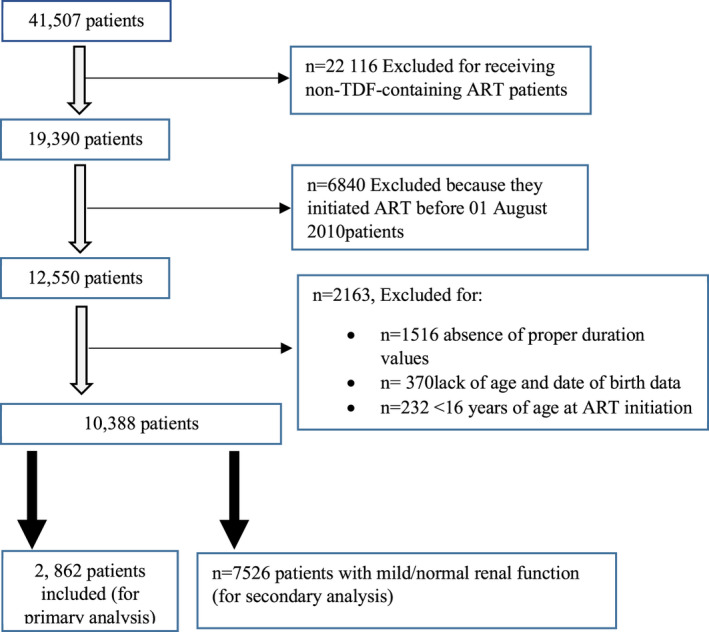
Selection of patients for the assessment of improvement and decline in CrCl

**Figure 2:**
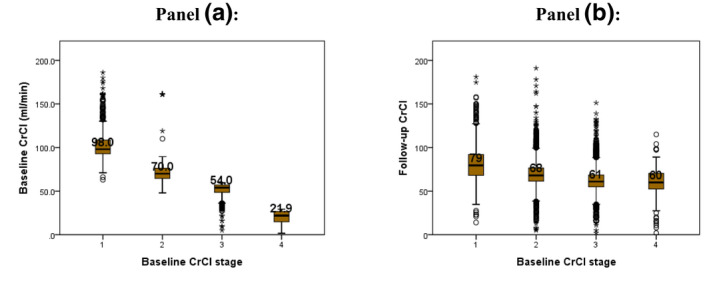
Baseline and follow‐up CrCl for patients according to stage of CrCl at baseline

A total of 10,388 patients were included in the study (Figure 1). They were divided into two groups based on the baseline CrCl. Group‐I consisted of patients with a baseline CrCl < 60 mL/min (*n* = 2862, 27.5%), and group‐II consisted of patients with a baseline CrCl ≥ 60 mL/min (n = 7526, 72.5%). Most were female (67.6%), and this was the case for both groups, although the proportion of females in the group‐I was significantly greater than in group‐II (Table 1). The cohort's mean (SD) age was 38 years, but the mean age of the first group was approximately three years younger than the second group (*P* < .001). The mean weight of the cohort was 63.7 kg, and no difference was found between the groups (*P* = .631). The mean CrCl at baseline was 63.2 ml/min, but the first group's mean baseline CrCl was significantly lower than that of the second group (*P* < .001). The duration of follow‐up was 2.2 years, and there was no difference between groups (*P* = .326). The mean follow‐up CrCl was 67 mL/min, which was 3.8 mL/min above the baseline. Although the group‐I still had a significantly lesser CrCl than group‐II, their follow‐up CrCl was ~12 mL/min above their baseline CrCl, while for the second group the follow‐up CrCl was 4 mL/min lower than their baseline CrCl (Table 1). Approximately 39% had a poor HIV status, but group‐I had a significantly less proportion of these cases (*P* = .013). There were about 1.9% records of pregnancy, but group‐I had a significantly higher proportion than the second (*P* = .003). We found few cases of hepatitis‐B surface antigen, and there was no difference between the groups (*P* = .515). A sum of 13.5% had received isoniazid preventive therapy (IPT), but the first group had fewer cases than the second group (*P* = .017). Few patients (5.9%) were receiving ART regimens containing TDF and a protease inhibitor (PI), but there were fewer patients in the first group than the second group (*P* = .011), (Table 1). The general trend was an increase in CrCl for patients in the first group. The opposite was true for patients in the second group (Figure 2).

**Table 1:** Characteristics of the patients in the study, and changes in renal function

Variable	All patients (n = 10,388)	Moderate/severe group (n = 2862)	Mild/normal group (n = 7526)	*P*‐value
Age (years, mean (SD)	38.0 (9.3)	36.5 (9.1)	39.4 (9.4)	<0.001
weight (kg), mean (SD)	63.3 (13.8)	62.9 (14.0)	63.7 (13.6)	0.631
Duration of follow‐up (years), mean (SD)	2.2 (1.7)	2.1 (1.7)	2.2 (1.7)	0.326
Baseline CrCl (ml/min)	63.2 (12.0)	50.9 (8.7)	75.5 (15.2)	<0.001
Follow‐up CrCl	67.0 (14.5)	62.7 (13.7))	71.2 (15.2)	<0.001
Gender				
Female	7021 (67.6)	2402 (83.9)	4619 (61.4)	<0.001
Male	3367 (32.4)	460 (16.1)	2907 (38.6)	
HIV status marked with a CD4 count < 200 or VL > 1000 or TB or a combination of either				
Yes	3988 (39.0)	1044 (36.5)	2944 (39.1)	0.013
No	6400 (61.0)	1818 (63.5)	4582 (60.9)	
Pregnancy				
Yes	198 (1.9)	73 (2.6)	125 (1.7)	0.003
No	10 190 (98.1)	2789 (97.4)	7401 (98.3)	
Hepatitis‐B surface antigen positive				
Yes	55 (0.5)	13 (0.5)	42 (0.6)	0.515
No	10 333 (99.5)	2849 (99.5)	7484 (99.4)	
Used Isoniazid preventive therapy				
Yes	1398 (13.5)	348 (12.2)	1050 (14.0)	0.017
No	8990 (86.5)	2514(87.8)	6476 (86.0)	
Improvement in renal function (≥25% increase in CrCl)				
Yes	1678 (16.2)	1147 (40.1)	531 (7.1)	<0.001
No	8710 (83.8)	1715(59.9)	6995 (92.9)	
Any improvement renal function (≥3ml/min/year increase in CrCl)				
Yes	4305 (41.4)	2065 (72.2)	2240 (29.8)	<0.001
No	6083 (58.6)	797 (27.8)	5286 (70.2)	
Decline in renal function (≥25% decrease in CrCl)				
Yes	1040 (10.0)	65 (2.3)	975 (13.0)	<0.001
No	9348 (90.0)	2797 (97.7)	6551 (87.0)	
Any decline (≥3ml/min/year)				
Yes	3085 (29.7)	201 (7.0)	2884 (38.3)	<0.001
No	7303 (70.3)	2661 (93.0)	4642 (61.7)	
Receiving Ritonavir along with TDF				
Yes	608 (5.9)	140 (4.9)	468 (6.2)	0.011
No	9780 (94.1)	2722 (95.1)	7058 (93.8)	
Regression to lower stage of renal function				
Yes	1929 (18.6)	29 (1.0)	1900 (25.2)	<0.001
No	8459 (81.4)	2833 (99.0)	6626 (74.8)	
Regression to severe stage of renal function				
Yes	63 (0.6)	29 (1.0)	34 (0.5)	0.001
No	10 325 (99.4)	2833 (99.0)	7492 (99.5)	

CD4 count < 200 mm^3^, VL > 1000 copies/L and Tuberculosis.

**Figure 3:**
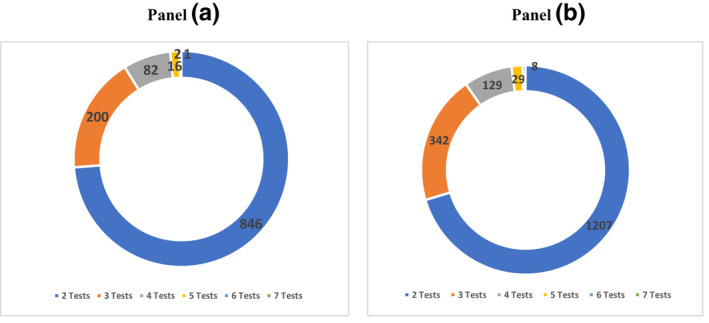
Distribution of patients in the improvement group (panel A) and non‐improvement group (panel B) according to the number of CrCl result recorded

**Figure 4:**
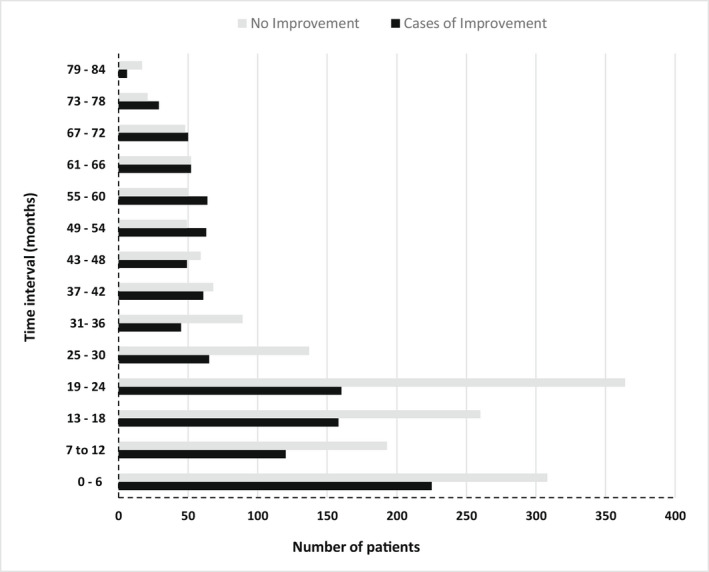
Test results distributed unequally across the time intervals

Of the 2,862 patients in the first group, improvement in CrCl occurred in 40.1% (n = 1147), who were referred to as the improvement group (Table 2). The rest formed the non‐improvement group. The non‐improvement group included patients who had > 3 mL/min/year increases in CrCl for who the follow‐up CrCl was < 25% above the baseline CrCl, and those who experienced any decline in CrCl. Most patients had two CrCl records (Figure 3: panels A and B, for improvement and non‐improvement groups, respectively). The follow‐up CrCl results were spread across time‐intervals (Figure 4). The improvement group were older (*P* = .015); had a longer duration of follow‐up (*P* < .001), a lower baseline CrCl (*P* < .001), a greater proportion of male patients (*P* < .001), and less pregnancy events than the non‐improvement group (*P* < .001) (Table 2). Furthermore, the improvement group had non‐significantly higher proportions of patients with a CD4 count < 200 cells/mm^3^, a viral load > 1000 copies/L, and non‐significantly lower proportions with tuberculosis (TB), which culminated in a greater proportion of patients with a poor HIV‐status than the non‐improvement group (*P* = .032) (Table 2.) The follow‐up mean CrCl of the improvement group was higher than their baseline by 26.0 ml/min, while for the non‐improvement group it was 2.3 ml/min higher than their baseline. The follow‐up CrCl of the improvement group was significantly greater than that of the non‐improvement group (Table 2). The sub‐analysis, which included 1788 patients, showed that the mean duration of follow‐up was significantly shorter for the improvement group than for the non‐improvement group (Table 3): a deviation from the findings for the whole group (n = 2862), (Table 2).

**Table 2:** Factors associated with CrCl improvement in patients with baseline CrCl less than 60ml/min

Variable	Improvement group	Non‐improvement group	*p*‐value	Crude Odds Ratio (Confidence Interval)	*P‐*value	Adjusted Odds Ratio (Confidence Interval)	*P‐*value
Age (years, mean (SD)	37.1 ± 9.3	36.2 ± 8.9	0.015	1.0 (1.0 ‐ 1.0)^b^	0.009	‐	‐
weight (kg), mean (SD)	62.7 ± 13.7	63.0 ± 14.2	0.568	1.0 (1.0 ‐ 1.0)^b^	0.580	‐	‐
Duration of follow‐up (years), mean (SD)	2.3 ± 1.9	2.0 ± 1.6	<0.001	1.1 (1.06 ‐ 1.2)	<0.001	1.1 (1.06 ‐ 1.2)	<0.001
	***0.9 ± 0*.** 6^c^	***1.1 ± 0.6*** c	***<0.001***	***0.7 (0.6 ‐ 0.8)*** *d*		***0.7 (0.6 ‐ 0.8)*** c	
Baseline CrCl (ml/min)	46.0 ± 10.7	54.2 ± 5.0	<0.001	‐	‐	‐	‐
Follow‐up CrCl	72.0 ± 14.1	56.5 ± 9.2	<0.001	‐	‐	‐	‐
Gender, Male							
Yes	896 (78.2)	1505 (87.8)	<0.001	2.0 (1.6 ‐ 2.4) ***1.6 (1.2*** ‐ ***2.0)*** c	<0.001	1.8 (1.5 ‐ 2.2) ***1.4 (1.03 ‐ 1.8)*** c	<0.001
No	250 (21.8)	210 (12.2)		1.0 (reference)			
HIV status^a^							
Yes	451 (39.4)	607 (35.4)	0.032	1.2 (1.1 ‐ 1.4) ***1.5 (1.2*** ‐ ***1.8)*** c	0.010 *<0.001*	1.3 (1.1 ‐ 1.5) ***1.4 (1.2 ‐ 1.8)*** c	0.007 *<0.001*
No	695 (60.6)	1108 (64.6)		1.0 (reference)			
Stage IV CrCl							
Yes	82 (90.1)	9 (9.9)	<0.001	14.6 (7.3 ‐ 29.1)	<0.001	13.4 (6.7 ‐ 26.8)	<0.001
				***12.1 (5.7*** ‐ ***25.7)*** c	*<0.001*	***12.5 (5.8 ‐ 26.7)*** *d*	*<0.001*
No	1065 (38.4)	1706 (61.6)		1.0 (reference)			
Pregnancy							
Yes	18 (1.6)	54 (3.1)	0.008	0.5 (0.3 ‐ 0.9)	0.015		
No	1128 (98.4)	1661 (96.9)		1.0 (reference)			
Hepatitis‐B surface antigen positive							
Yes	6 (0.5)	7 (0.4)	0.652	1.3 (0.5 ‐ 3.8)	0.241	‐	‐
No	1140 (99.5)	1708 (99.6)		1.0 (reference)			
Used Isoniazid preventive therapy							
Yes	167 (14.6)	263 (15.3)	0.582	0.9 (0.7 ‐ 1.1)	0.323	‐	‐
No	978 (85.4)	1452 (84.7)		1.0 (reference)			
Receiving Ritonavir along with TDF							
Yes	64 (5.6)	76 (4.4)	0.161	1.4 (0.9 ‐ 2.0)	0.123	‐	‐
No	1082 (94.4)	1639 (95.6)		1.0 (reference)			
CD4 count < 200 cells/mm^c^							
Yes	191 (16.7)	248 (14.5)	0.109	‐	‐	‐	‐
No	955 (83.3)	1467 (85.5)					
Viral load > 1000 copies/L^d^							
Yes	246 (21.5)	322 (18.8)	0.077	‐	‐	‐	‐
No	900 (78.5)	1393 (81.2)					
Tuberculosis^d^							
Yes	111 (9.7)	172 (10.0)	0.763	‐	‐	‐	‐
No	1035 (90.3)	1543 (90.0)					
Improvement (≥25% increase in CrCl)	1146	n/a	‐	‐	‐	‐	‐
Any improvement (≥3ml/min/year increase in CrCl)	1146	582	‐	‐	‐	‐	‐
Any decline (≥3ml/min/year)	n/a	201	‐	‐	‐	‐	‐
Decline in renal function (≥25% decrease in CrCl)	n/a	65	‐	‐	‐	‐	‐
Regression to severe stage of renal function	n/a	28	‐	‐	‐	‐	‐

^a^ HIV status marked with a CD4 count < 200 or VL > 1000 or TB or a combination of either of these variable (Poor status = Yes);

^b^ means ‐figures were rounded off;

^c^ Sub‐analysis findings, which involved 62.5% of the patients (n = 1788);

^d^ variables marked with this symbol were not used in the model as they contributed to the HIV status variable. The italicised values are findings generated from the sub‐analysis.

**Table 3:** Sub‐analysis: Duration of follow‐up for the improvement group vs. non‐improvement group based on different time‐points

Duration of follow‐up		Number of patients		*P*‐value (t‐test)	Duration of follow‐up: mean (SD), years		F‐value	*P*‐value (F‐test)	*P*‐value (t‐test)
		Improvement	Non‐improvement		Improvement	Non‐improvement			
0‐6 months	Female	265 (58.6)	187 (41.4)	.352	0.22 (0.15)	0.3 (0.2)	0.099	.753	.012
	Male	43 (53.1)	38 (46.9)						
7‐12 months	Female	174 (63.7)	99 (36.3)	.049	0.8 (0.2)	0.8 (0.2)	2.186	.140	.540
	Male	19 (47.5)	21 (52.5)						
13‐18 months	Female	241 (63.9)	136 (36.1)	.027	1.3 (0.2)	1.3 (0.2)	0.024	.876	.994
	Male	19 (46.3)	22 (53.7)						
19‐24 months	Female	308 (71.5)	123 (28.5)	.033	1.7 (0.1)	1.7 (0.1)	0.792	.374	.956
	Male	56 (60.2)	37 (39.8)						
**0‐24 months**	**Female**	**988 (64.4)**	**545 (35.6)**	**.001**	**0.9 (0.6)**	**1.1 (0.6)**	**1.169**	**.280**	**<.001**
	**Male**	**137 (53.7)**	**118 (46.3)**						

Note

More male patients experienced improvement at all time‐ intervals except the first one, where no difference in proportions was observed. The duration of follow‐up was significantly shorter for the patients who experienced improvement than for the corresponding group. Most patients in the improvement group had the second test results recorded after 24 months, widely spread across the time intervals. The event of improvement could not be traced to a specific time interval. For this reason, they were not included in the sub‐analysis.

Multivariate binary logistic regression analysis for the 2862 patients showed that male patients were 1.8 times more likely to experience improvement in CrCl than females (*P* < .001); that patients who were in stage IV of CrCl, were 13.4 times more likely to experience improvement than patients who were in stage III of CrCl (*P* < .001); that for every additional year of ART, the odds of experiencing improvement in CrCl were 1.1 (*P* < .001); and that patients who were in a poor HIV status, were 1.2 times more likely to experience improvement than their counterparts (*P* < .001), (Table 2). The regression analysis was repeated for the sub‐group, which generated similar results for all predictor variables, except duration of follow‐up for which the effect was reversed: from an OR of 1.1 to 0.7.

The proportion of events of 25% decline in CrCl among the < 60 mL/min group was lower than the corresponding proportion in the ≥ 60 mL/min group (*P* < .001) (Table 2). Similarly, the transition to lower stages of CrCl in the < 60 ml/min group was lower than in the ≥ 60 mL/min group: 1.0% vs. 25.2%; *P* < .001, (Table 2 ). Nonetheless, more patients from the < 60 mL/min group than from the ≥ 60 mL/min group transitioned to CrCl stage IV (Table 2 ).

## 4 DISCUSSION

This in‐depth assessment of the effects of TDF‐containing ART on renal function in HIV‐positive patients with a baseline CrCl of < 60 mL/min, involved two referral hospitals and included many patients. We found that a large proportion of patients experienced improvement in CrCl following the initiation of ART (TDF‐containing). The factors that were associated with improvement in CrCl included stage IV of CrCl at baseline, male gender, and a poor HIV status. We found that decline in CrCl was more frequent among patients with a baseline CrCl ≥ 60 mL/min than in those with a baseline CrCl < 60 mL/min. We found that disproportionately more patients in the ≥ 60 ml/min group transitioned to lower stages of CrCl; however, disproportionately fewer patients in the ≥ 60 ml/min group than in the < 60 mL/min, transitioned to stage IV of CrCl.

Our study documents a significantly greater proportion of patients experiencing improvement than decline in CrCl following the initiation of ART, moreover they received TDF‐containing ART. These findings agree with a previous study we conducted on fewer patients.^17^ Mulenga *et al,* observed improvement in renal function for patients with severe renal impairment at baseline, even with TDF‐containing ART.^8^ We found that improvement in CrCl was an early event, and this has been implied by Mpondo *et al* and Kalayjian.^7^, ^28^ In our study, most patients had two renal function test results including the baseline and the second test, but the second test results were spread across various time intervals making it a challenge to assess the effect of duration of exposure to the outcome of interest. The sub‐analysis we conducted to sort out this challenge exposed an interaction bias, as the direction of effect was reversed. The latter finding was more likely than the former because we found improvement within the first year for most patients who had more than two renal function test results. Since renal impairment is a complication of HIV, the improvement we observed suggests that some patients in the < 60 mL/min group were experiencing HIV‐related renal impairment, which responds to highly active ART.^29^ This preposition leans on the fact that the likelihood of experiencing improvement among patients in CrCl stage IV at baseline was several folds that of the patients who were in stage III of CrCl. HIV is known to cause HIVAN, HIV immune complex kidney disease, and opportunistic infections in the parenchyma of the kidney, which affect kidney function.^6^, ^30^, ^31^, ^32^, ^33^, ^34^ Currently, an alternative antiretroviral drug to TDF has been recommended for patients with a CrCl < 60 mL/min as a precautionary measure against further decline.^13^ However, the available alternatives, namely zidovudine (AZT) and Stavudine (D4T), are associated with severe adverse events that have led to their withdrawal from the preferred ART regimens, in favor of TDF; and sometimes they are contraindicated. For example: AZT is contraindicated in patients with severe anemia at baseline.^35^ Furthermore, the significantly greater improvement than decline we observed, suggests that the prescription of TDF for patients with a CrCl < 60 mL/min was clinically logical,^7^, ^29^ and careful matching of the dose with the CrCl should be ensured. Close monitoring of renal function in these patients may be necessary, due to TDF‐associated nephrotoxicity,^18^, ^36^ and bearing in mind that the period of exposure was relatively short. Also, comorbidities that may be associated with further decline should be investigated prior to initiating TDF‐containing ART. We could not explain why male gender was associated with a significantly greater proportion of improvement events, but it could be a result of disturbance in the female patients’ hormonal milieu, resulting in hypoestrogenemia and loss of estrogen's renal protective effects.^37^ Furthermore, regarding male gender, their older age may have influenced the age variable making older age a predictor of improvement. This is unlikely, because increase in age is associated with a reduction in the number of functional nephrons.^38^ A separate analysis on male patients showed that older age was not a predictor for improvement. A similar analysis on female gender supported the association between increasing age and improvement, and this increased the likelihood of an unknown confounding variable.

TDF is etiologically related to nephrotoxicity,^39^, ^40^ and mechanisms of TDF‐associated nephrotoxicity have been suggested.^41^, ^42^ Nonetheless, through this study we endeavor to answer the question ‘what is the incidence rate’, and the question ‘who is at risk regarding the baseline CrCl?’ This is important because of conflicting evidence regarding both incidence and risk factors,^8^, ^9^, ^43^, ^44^ and because the findings pertain to sub‐Saharan Africa, where there is a low research output, except in South‐Africa.^45^ Regarding the question on incidence rate, our study has shown that TDF‐associated nephrotoxicity per‐se is common, but not all events were clinically significant. Regarding the question ‘who is at risk’, our study has shown that patients with a baseline CrCl ≥ 60 mL/min were at a greater risk of experiencing the decline compared with those who had < 60 mL/min, and that not all declines were clinically important. Therefore, the more important outcome to study would be a transition to stage IV of CrCl, including end‐stage‐renal‐disease, rather than decline in CrCl. A study by Ojeh *et al* put the incidence of TDF‐associated renal impairment at 4.6%,^46^ but did not provide the proportion who migrated to the severe stage of renal function. Brennan *et al* reported that patients with a moderate renal function at baseline were more at risk of nephrotoxicity than others.^47^ Our findings agree with Brennan *et al* regarding the risk; but the proportion was too low to undermine the benefits of TDF‐containing ART. The incidence of new onset severe reduced CrCl in the ≥ 60 mL/min group in this study (0.5%) is similar to findings by Kyaw *et al* (0.3%), Winston *et al* (0.43%) and Nishijima *et al* (0.5%).^48^, ^49^, ^50^ Our study puts the incidence of new onset severe reduced CrCl at 1.0% among patients with a CrCl in the moderate CrCl stage, at baseline. Further investigation of this that deals with confounders such as diabetes mellitus and hypertension can generate better results.^51^ Improvement for female patients being significantly lower than that of the males could be due to HIV‐associated disturbances in the hormonal milieu among the female patients, resulting in ovarian insufficiency. The resultant hypoestrogenemia is associated with loss of the renal protective effects of estrogen.^52^, ^53^ The findings of this study support the use of TDF‐containing ART in patients with moderately or severely reduced CrCl at baseline, particularly in patients without co‐existing diseases known to affect renal function.

The findings of this research provide a solution to the clinician caring for an individual ART naïve HIV‐positive patient with a low CrCl. The patient may be coinfected with hepatitis B thus needing TDF‐containing ART^13^, ^54^ or may have attending clinical states rendering the use of alternatives to TDF contraindicated. Our study supports the use of TDF in these patients; however, some patients may have co‐morbidities that increase their risk of experiencing nephrotoxicity.^55^ Therefore, the decision to use TDF‐containing ART should be individualized. Careful attention should be given to TDF’s dose, matching it with the increasing CrCl. Furthermore, the clinician should set one's attention on the estimated CrCl rather than the magnitude of reduction, since some large reductions in CrCl never required changes in dose or dosage intervals. Individualization of therapy falls under clinical pharmacology. Therefore, this research is suitable for publication in this journal.

Our study had limitations. Regarding improvement, the definition there of was restrictive. As a result, a substantial number of patients who experienced increases in CrCl were not included among the improvement group, and may infringe on the benefits of TDF‐containing ART. We conducted a separate analysis including these patients, which generated the same results concerning the predictors. Another limitation was the various and irregular periods of follow‐up, which made it difficult to assess specific periods such as at six and twelve months, for many patients. In response, we conducted an assessment based on time intervals, for the patients whose follow‐up renal function test results were conducted within two years, including those who had more than two test results yielding similar results. Regarding decline in CrCl our study was limited by the lack of objective evidence to explain why the declines occurred in the < 60 mL/min group. Nonetheless, TDF has the propensity to cause a significant decline in CrCl, and a synergistic interaction between TDF and co‐existing non‐HIV related risk factors are known etiologies for the decline in CrCl.^41^, ^46^, ^56^, ^57^, ^58^

## 5 CONCLUSION

The initiation of TDF‐containing ART in patients with a baseline CrCl < 60 mL/min was associated with significantly more patients experiencing improvement than decline. The study suggests that patients who have HIV‐associated renal disease are more likely to improve with ART, including TDF‐containing regimens. Improvement should be expected to occur within the early period of ART. Male gender was also a factor associated with more improvement than female gender. Decline in CrCl should also be expected but in a rather small proportion of patients who had a CrCl < 60 mL/min at baseline. Careful monitoring may still be required since some of the declines may culminate in new onset severe reduced CrCl. Overall, this study suggests that TDF‐containing ART may be used in patients with a baseline CrCl < 60 mL/min. Although it was not part of this study, it is worth mentioning that prior to initiating TDF‐containing ART, the patients should be investigated for comorbidities that are known to increase the risk of decline in CrCl.

## 6 Data Availability Statement

The data that were used in this study may not be readily available as it may compromise patient confidentiality.

## Author contribution

Presented below are the contributions of the authors to the study presented in this manuscript.

- **FK:** Concept, study design, data analysis, manuscript
- **BG:** Manuscript review
- **AS:** Concept, study design, manuscript review
- **TR:** Concept, study design, manuscript review
